# Interleukin-6, tumor necrosis factor-alpha and receptor activator of nuclear factor kappa ligand are elevated in hypertrophic gastric mucosa of pachydermoperiostosis

**DOI:** 10.1038/s41598-017-09671-7

**Published:** 2017-08-29

**Authors:** Hui Huang, Yongjun Wang, Yong Cao, Boda Wu, Yonggui Li, Liangliang Fan, Zhiping Tan, Yi Jiang, Jianguang Tang, Jianzhong Hu, Xiaoliu Shi

**Affiliations:** 1 Department of Medical Genetics, The Second Xiangya Hospital, Central South University, Changsha, Hunan 410011 China; 2 Department of Gastroenterology, The Second Xiangya Hospital, Central South University, Changsha, Hunan 410011 China; 3 Department of Spine Surgery, Xiangya Hospital, Central South University, Changsha, Hunan 410008 China; 4 The Thoracic Surgery Research Room, The Second Xiangya Hospital, Central South University, Changsha, Hunan 410011 China; 5 Department of Pathology, The Second Xiangya Hospital, Central South University, Changsha, Hunan 410011 China; 6 Department of Neurology, The Second Xiangya Hospital, Central South University, Changsha, Hunan 410011 China

## Abstract

Pachydermoperiostosis (PDP) is a rare inherited multisystem disease characterized with digital clubbing, pachydermia and periostosis. Variants in either *HPGD* or *SLCO2A1* that interrupt the prostaglandin E2 (PGE_2_) pathway have been shown to be involved in PDP. Here, in addition to six confirmed variants in *HPGD* or *SLCO2A1*, we identified four novel *SLCO2A1* variants in eight PDP patients from seven Chinese Han families. In addition, gastric mucosa hyperplasia was observed in all affected individuals and interleukin-6 (IL-6), tumor necrosis factor-alpha (TNFα) and receptor activator of nuclear factor kappa ligand (RANKL) expression were elevated in hypertrophic gastric mucosa. Two of eight patients who had severe arthralgia were treated with celecoxib. After three months, their arthralgia was partly relieved and IL-6, TNFα and RANKL expression were decreased in accordance with their relieved hypertrophic gastric mucosa. Our study broadens the variation spectrum of *SLCO2A1* and suggests that the gastric mucosa hyperplasia might be a common characteristic of PDP. Moreover, celecoxib would be a considerable choice for PDP patients. We also revealed that IL-6, TNFα and RANKL may play important roles in the molecular mechanisms of gastric mucosa hyperplasia in PDP for the first time.

## Introduction

Pachydermoperiostosis (PDP), also named primary hypertrophic osteoarthropathy (HOA), is a rare genetic disease, whose major manifestations include digital clubbing, periostosis and pachydermia. Other clinical characteristics, such as arthralgia, arthritis, seborrhea, acne, hyperhidrosis, cutis verticis gyrate (CVG), acroosteolysis and ptosis were also reported^1, 2^. So far, variants in two genes involved in prostaglandin catabolic pathway, *HPGD* (MIM 259100)^3^ and *SLCO2A1* (MIM 614441)^4^, have been found to cause PDP. *HPGD* encodes the degradative enzyme 15-hydroxyprostaglandin dehydrogenase (15-PGDH), a critical enzyme of metabolizing prostaglandins^5^. And *SLCO2A1* encodes the prostaglandin transporter (PGT) that uptakes prostaglandins into cellular matrix for further degradation^6, 7^.

Most of the previous studies focused on bone and skin changes of PDP patients, while their gastric conditions were seldom reported. In fact, most PDP patients did not receive endoscopy or other relative examinations so that their gastric lesions might be ignored. Still, relative symptoms have been repeatedly reported by different groups independently. Lakshmi TS *et al*. first put forward that a PDP patient was associated with Menetrier’s disease^8^, a rare disease characterized by massive overgrowth of mucous cells (foveola) in the mucous membrane lining the gastric, resulting in large gastric folds. M.BHARATH *et al*. also reported a PDP patient associated Menetrier’s disease in 2016^9^. Three independent reports showed hypertrophic gastric mucosa in PDP patients and gastric biopsy revealed gastric mucosa hyperplasia of the surface epithelium, cystic changes, stromal edema with inflammatory cells infiltration^10–12^. Other three PDP patients with hypertrophic gastritis were also respectively reported but they didn’t receive histological examination^13–15^. In addition, digestive system disease, such as ulcer, juvenile polyps, gastric cancer, Crohn’s disease and protein-losing enteropathy, were also reported occurring as occasional associations of PDP^16–18^. Early in 2001, Zrinka Jajic *et al*. performed gastroscopy examination in 21 of 76 PDP patients (27.6%) and they found all of these patients had ulcer or hypertrophic gastritis^19^. In 2011, according to Fortes BC’s summary, more than 20% PDP patients present gastric system abnormities^20^.

The specific pathogenesis of PDP is still unclear. Besides the PGE_2_ metabolism, inflammation and unbalance between the bone resorption and osteogenesis also play important roles in PDP^3, 4, 7^. Several cytokines and cell receptors, such as interleukin-6 (IL-6), tumor necrosis factor-alpha (TNFα), receptor activator of nuclear factor Kappa ligand (RANKL), were reported increasing in serum and synovial fluid of PDP patients^21–23^. We detected the IL-6, TNFα and RANKL expression in hypertrophic gastric mucosa of PDP patients for the first time.

## Patients and Methods

### Patients and Ethics

**Figure 1:**
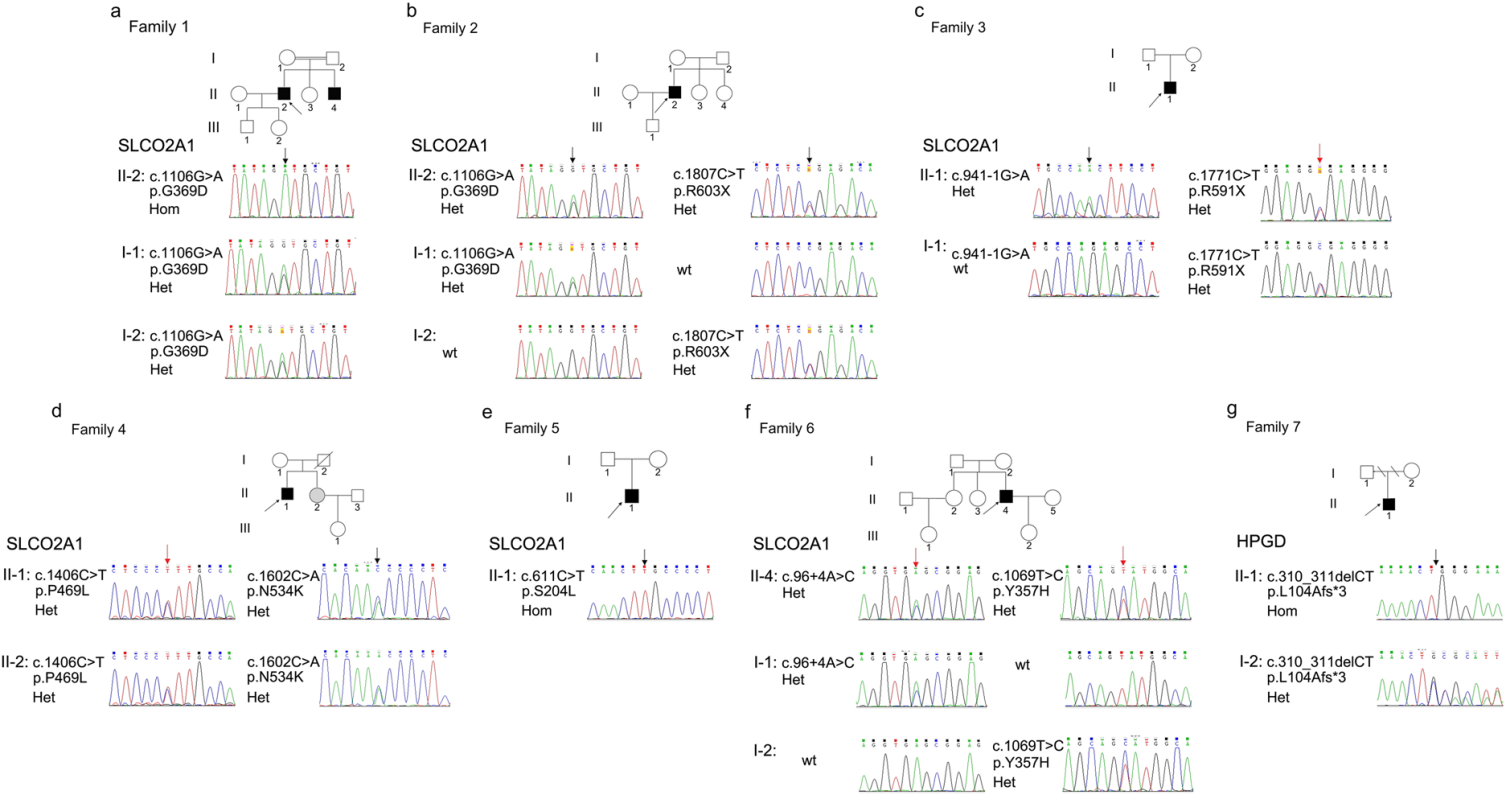
Pedigrees and variants of seven families affected by pachydermoperiostosis. The arrows in pedigrees show probands in the families. Black symbols mean PDP patients. Gray symbols mean unaffected homozygous or compound heterozygous carriers. Red arrows show novel variants on *SLCO2A1* and black arrows show reported variants on *SLCO2A1* and *HPGD* in chromatograms. Het, heterozygous. Hom, homozygous. Wt, wild type.

This study was approved by the Ethics Committee of The Second Xiangya Hospital of Central South University and all methods were in accordance with the Declaration of Helsinki. All participants have provided informed written consent to genetic and biochemical analysis and to publication of all identifying images. All PDP patients were of Han ethnicity and were diagnosed using established clinical and radiological criteria as a standard^1^. The pedigrees of these seven families are shown in Fig. 1. All patients were born from healthy non-consanguineous couples except patient1 and patient2, who were born from second-cousin unaffected parents. (Fig. 1a) Parental material of patient5 and patient6 and maternal material of patient4 were not available, as well as father’s material of patient8.

### Variant analysis

Genomic DNA was extracted from peripheral white blood cells using the QIAamp DNA Blood Maxi Kit (QIAGEN KK, Tokyo, Japan). The whole DNA sequence of *HPGD* and *SLCO2A1* were obtained from the available online database (UCSC: NM_00860.5, NM_005630.2). PCR was performed to amplify all exons and exon-intron boundaries of *SLCO2A1* and *HPGD* followed by direct sequencing (Primers seen in Table S1). Online software, Mutationtaster (http://www.mutationtaster.org)^24^, PolyPhen-2 (http://genetics.bwh.harvard.edu/pph2/)^25^, and SIFT (http://sift.jcvi.org/)^26^ were used to predict the damaging effect of missense variants, and the HSF(http://www.umd.be/HSF3/)^27^ was used to analyze the damaging effect of splicing site variants. The pathogenicity of variants was interpreted according to ACMG guidelines^28^.

### Immunohistochemical staining

8 PDP patients and 4 relative normal controls’ paraffin embedded gastric mucosa tissues were performed immunohistochemical staining. Specimens were grilled for 2 hours at 60° C oven at first. Then the specimens were dewaxed with xylene and rehydrated with gradient alcohol. Antigen retrieval was performed by using 0.01 mmol citrate buffer to make the samples infiltrate in for 15 minutes after steaming in pressure-cooker. After retrieval, the tissues were dealt with rabbit antibody against human IL-6 (abcam, ab9324) and mouse antibody against human RANKL (abcam, ab45039) and TNFα (abcam, ab6671) overnight at 4 °C. Biotinylated secondary antibody was adopted in SP kit at room temperature for 10 minutes followed with incubating of an appropriate amount of HRP-conjugated secondary antibody at room temperature for 30 minutes. Immunostaining was carried out through using DAB for chromogen and hematoxylin for nuclear staining. The specimens were cleared in xylene after dehydrated with gradient alcohol. Finally the neutral beams were applied to seal the specimens. All immunostained specimens were examined with the microscope (BA210T, Motic) and assessed by two pathologists blinding to clinical features. Under the Microscopy, the images and IOD (integrated optical density) were acquired with the Image-Pro Plus software 6.0.

## Results

### Clinical manifestations

All eight patients are males and began to show symptoms of PDP before their age of 20. Their major clinical manifestations were summarized in Table 1 and all of them were diagnosed as major PDP. Patient4, the propositus in family3, developed swollen knee joints gradually which finally lead to severe motion limitation at the age of 24 (Fig. S1c). Patient8 does not have diaphyseal periostosis but shows distinctive features clubbing of fingers and toes and acroosteolysis (Fig. S1d,e). Other members of these seven families are normal except patient5’s sister who received subtotal gastrectomy because of peptic ulcer when she was 24 years old.

**Table 1:** The clinical characteristics of patients with pachydermoperiostosis.

*Patient*		P1	P2	P3	P4	P5	P6	P7	P8
*gender*		Male	Male	Male	Male	Male	Male	Male	Male
*current age,y*		32	27	33	24	33	33	32	16
*onset age,y*		19	18	21	15	19	14	16	12
*skeletal changes*									
	*Clubbing of finger and toes*	+	+	+	+	+	+	+	++a
	*diaphyseal Periostosis*	+	+	+	+	+	+	+	−
	*Acroosteolysis*	−	−	−	−	−	−	−	+
	*swollen joints*	+	+	+	++b	+	+	+	+
	*arthralgia*	+	+	+	+	+	+	+	+
	*limitation of motion*	−	+	+	++	+	+	+	+
*Skin*									
	*Pachydermia*	++	++	+	+	+	+	+	+
	*Hyperhidrosis*	+	+	+	+	+	+	+	+
	*Cutis gyrate*	++	++	++c	++	++	++	++	−
	*Seborrheic hyperplasia*	+	+	+	+	+	+	+	+
	*acne*	+	+	+	+	+	+	+	++
*Gastric mucosa hyperplasia*	*endoscopy tests*	+++d	+++	++	++	+	+	++	+++
	*histological examination*	+++	+++	++	++	+	+	++	+++

P, patient; −, negative; +, positive; ++, obvious performance; +++, severe condition; a, digital clubbing is obviously; b, a remarkable swollen knee joints; c, patient3 performed a facial plastic surgery when he was 26 years old; d, a very severe thickness of gastric mucosa.

**Figure 2:**
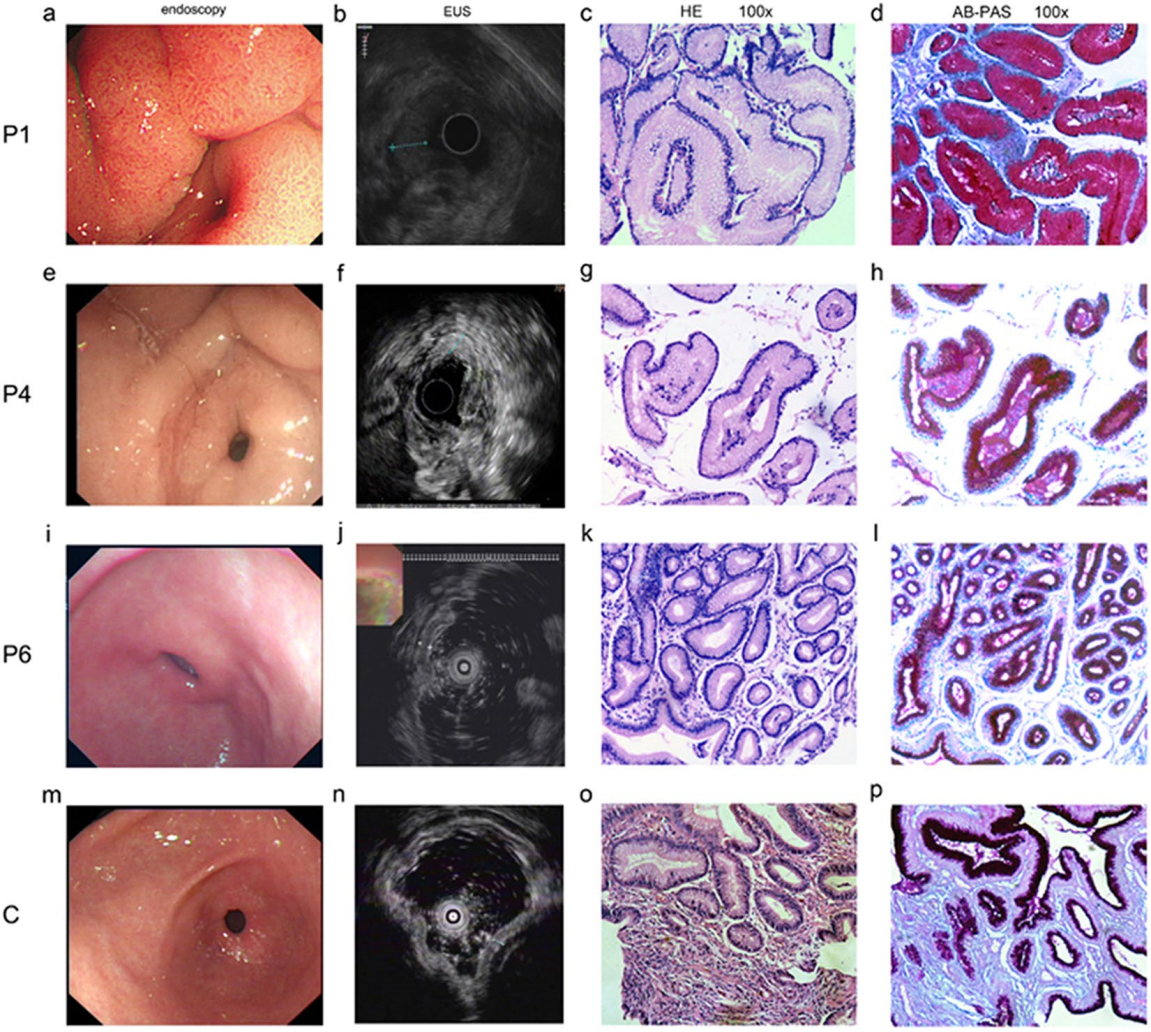
Representative clinical and histological manifestations of gastric mucosa hyperplasia in PDP patients. EUS, endoscopic ultrasonography. P, patient. C, control, a patient with mild chronic superficial gastritis. HE, hematoxylin eosin staining. AB-PAS, alcian blue-periodic acid stiff staining. Endoscopy and EUS results as well as HE staining and AB-PAS staining of gastric mucosa of patitent1 (**a–d**), patient4 (**e–h**), patient6 (**i–l**) and control (**m–p**). Patient1 (**a, b**), patient4 (**e, f**) and patient6 (**i, j**) present severely, moderately and mildly thickened gastric mucosa respectively. In consistence, gastric mucous glands hyperplasia, intracellular mucus increase and inflammation from patient1 (**c, d**), patient4 (**g, h**) and patient6 (**k, l**) reduced gradually. All the images and materials were originated from gastric antrum. Pathological diagnosis was provided by professional pathologist.

Endoscopy and endoscopic ultrasonography (EUS) were also performed on all patients. A patient with mild chronic superficial gastritis (Fig. 2 C) was also tested as a control. According to WE Bolch, the thickness of normal gastric mucosa is about 1.0–1.6 mm^29^. The control had a gastric mucosa of 1.8 mm. Comparing with the control, all PDP patients had similarly or more thickened gastric mucosa (Fig. 2): Patient1, patient2 and patient7, in particular, presented severely rough, thickened and gyrus-like gastric mucosa (9.1 mm, 4.6 mm and 5.6 mm respectively); Patient3, patient4, and patient8 showed moderately rough and thickened gastric mucosa (3.9mm, 3.8 mm, and 4.2 mm respectively); Patient5 and patient6 (2.3 mm and 2.4 mm respectively) had mildly thickened gastric mucosa comparable to that of the control. Furthermore, histological examination was employed to detect the pathologic changes in patients. Consistent with the results of endoscopy, all patients had different degrees of gastric mucosa hyperplasia. The patients with most thickened gastric mucosa showed most severe gastric mucous glands hyperplasia, intracellular mucus increase and inflammation (Fig. 2 P1 a–d). The inflammation and mucous glandular proliferation were reduced in moderately thickened gastric mucosa patients (Fig. 2 P4 e–h) and least severe in mildly thickened patients (Fig. 2 P6 i–l).

### Variants identification in *HPGD* and *SLCO2A1*

Employing PCR followed by direct sequence analysis, we detected variants in both *HPGD* and *SLCO2A1* in all patients. All detected variants were shown in Fig. 1 and Table S2. Five confirmed variants, four novel variants in *SLCO2A1* (Fig. 1a–f) and one reported variant in *HPGD* (Fig. 1g) were found. Among the four novel variants, there were two missense variants c.1069 T > C (Fig. 1f) and c.1406 C > T (Fig. 1d), a nonsense variant c.1771C > T (Fig. 1c) and an adenine-to-cytosine transition at the invariant + 4 position of the acceptor site of intron1 (c.96 + 4 A > C) (Fig. 1f). Since parents’ materials of patient5, patient6 and maternal materials of patient4 and paternal materials of patient8 were not available, we would not be able to get their genetic information. From our current data, all the variants found in patients were inherited from their normal parents. According to the ACMG guidelines^28^, all these variants are classified to be either pathogenic or likely pathogenic except c.96 + 4 A > C, which is of uncertain significance. Noticeably, patient5 and his little sister (Fig. 1d II-2) share the same compound heterozygous variants, she received subtotal gastrectomy at 24 years old because of peptic ulcer which was also reported as an association of PDP^16^. But she did not show any signs of skin and bone changes of PDP so far. The incomplete penetrance of PDP symptoms in females is observed repeatedly^30, 31^ and appeared to be significant. Currently, the underlying mechanism is not so clear. And previous researches suggested that it might be the crosstalk between estrogen and prostaglandin signal pathway^32, 33^. Other individuals in these families were heterozygous carriers or had no variants.

### IL-6, TNFα and RANKL expression were increased in hypertrophic gastric mucosa of PDP patients

**Figure 3:**
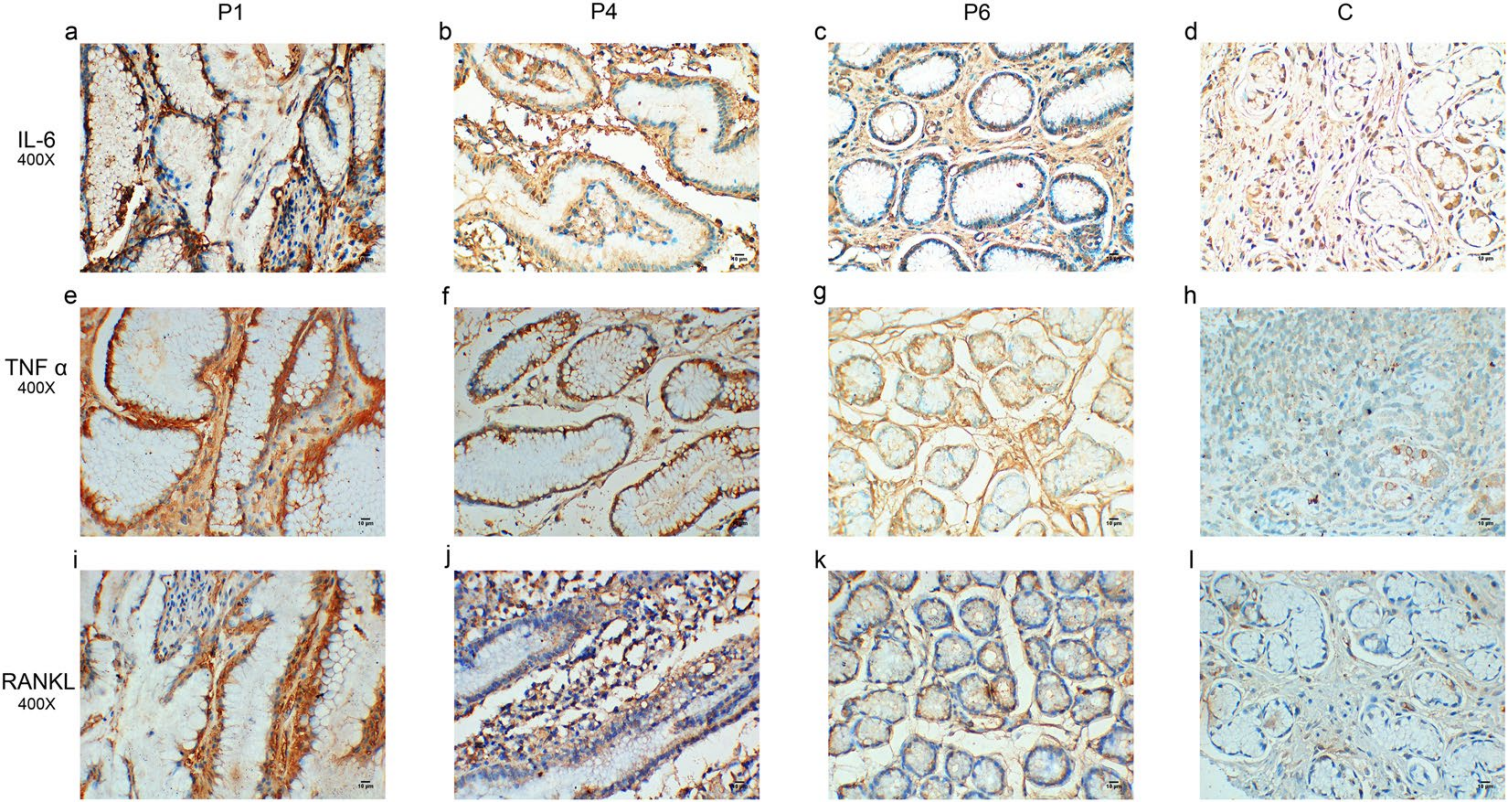
IL-6, TNFα and RANKL expression were increased in the hypertrophic gastric mucosa of PDP patients. P1, patient1; P4, patient4; P6, patient6; C, normal control. **a–d**, **e–h** and **i–l** showed immunohistochemistry staining of IL-6, TNFα and RANKL respectively. IL-6, TNFα and RANKL expression were stronger in hypertrophic gastric mucosa of PDP patients (P1 **a,e,i**, P4 **b,f,j**, P6 **c,g,k**) than that of healthy control (C **d,h,l**). IL-6, TNFα and RANKL expression were strongest in patient1 and gradually decreased in patient4 and patient6. Immunostained specimens were assessed by pathologists blinding to clinical features.

The expression of IL-6, TNFα and RANKL was detected in eight PDP patients and four normal controls. As Fig. 3 shows, IL-6, TNFα and RANKL expression were elevated in hypertrophic gastric mucosa of PDP patients (Fig. 3, P1, P4, P6) compared with normal control (Fig. 3C). And the differences in IOD between patients and controls were statistically significant (Fig. S2). In addition, in accordance with the severity of the hyperplasic gastric mucosa of eight PDP patients, IL-6, TNFα and RANKL expression were strongest in serious hypertrophic gastric mucosa (Fig. 3 P1 a e i), then gradually reduced in moderately (Fig. 3 P4 b f j) and mildly (Fig. 3 P6 c g k) thickened gastric mucosa.

### Celecoxib treatment was considered to be effective for PDP patients

**Figure 4:**
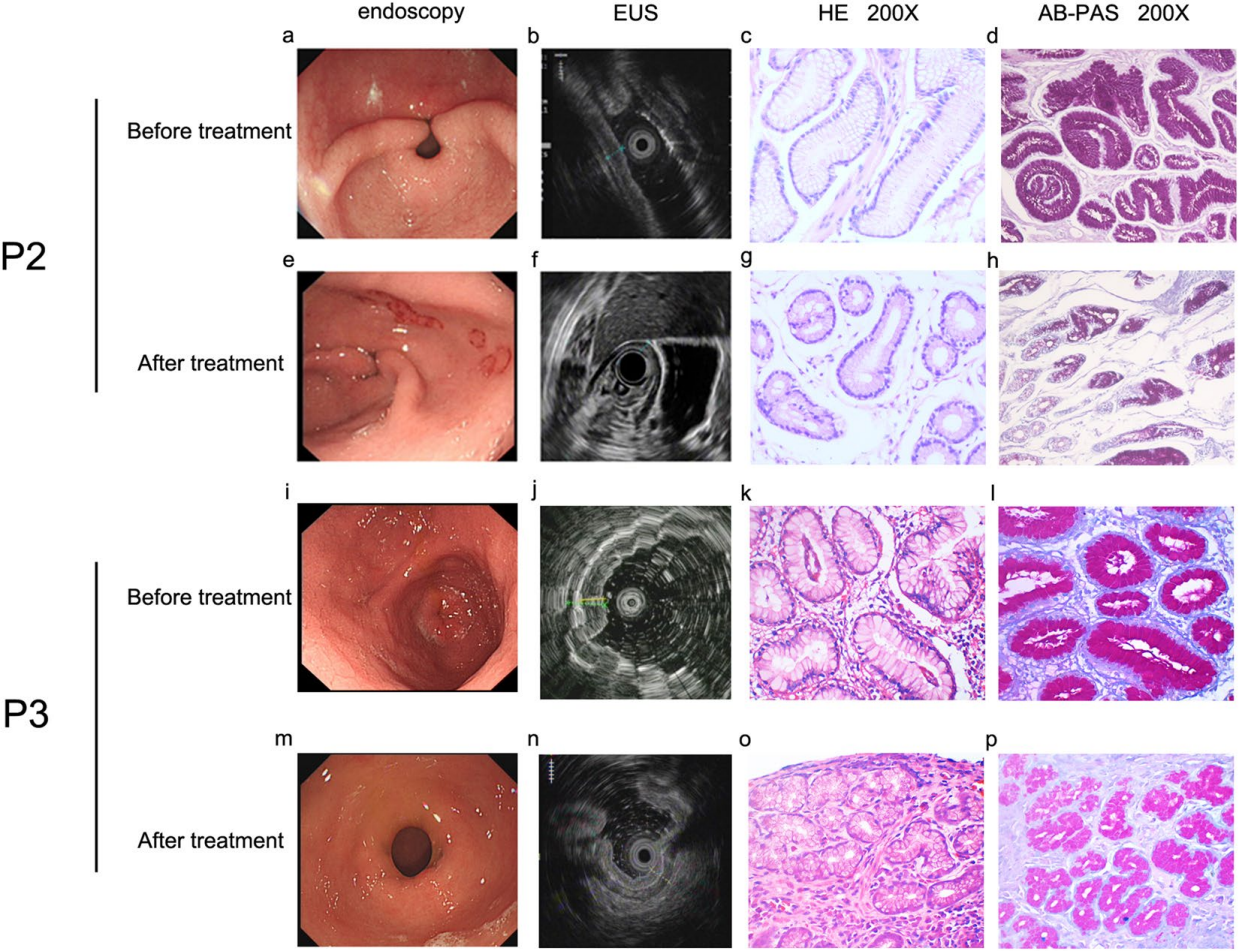
Changes of endoscopy and pathological features of patient2 and patient3 after them accepting celecoxib treatment. EUS, endoscopic ultrasonography. HE, hematoxylin eosin staining. AB-PAS, alcian blue-periodic acid stiff staining. P2, patient2. P3, patient3. Patient2 and patient3 were treated with celecoxib for 3 months. The effect of the treatment was shown by endoscopy (**a,e,i,m**) and endoscopic ultrasonography (**b,f,j,n**) as well as HE staining (**c,g,k,o**) and AB-PAS staining (**d,h**,**l,p**). The thickness of gastric mucosa declined after treatment (**e,f**,**m,n**). Consistently, patients showed inflammation, mucous glandular proliferation and intracellular mucus increase before treatment (**c,d**,**k,l**) and all these symptoms were reduced by treatment, but glandular proliferation still exist (**g,h,o,p**). Pathological diagnosis was provided by professional pathologist.

**Figure 5:**
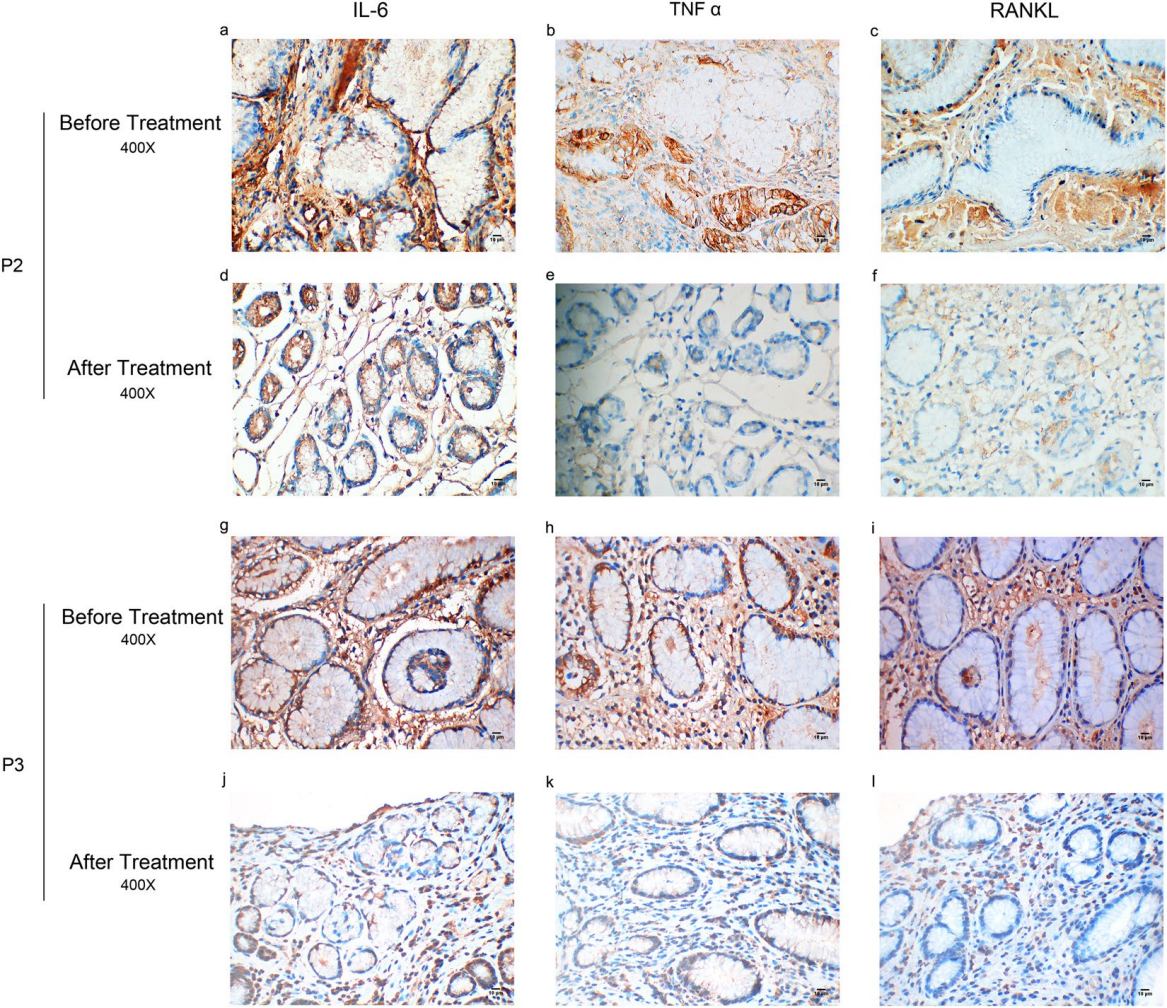
IL-6, TNFα and RANKL expression decreased in gastric mucosa of patient2 and patient3 after them receiving celecoxib treatment. P2, patient2; P3, patient3. (**a,d,g** and **j**) showed immunohistochemical staining of IL-6. (**b,e,h** and **k**) showed immunohistochemical staining of TNFα. (**c**,**f,i** and **l**) showed immunohhistochemical staining of RANKL. Patient2 and patient3 received celecoxib treatment for three months. IL-6, TNFα and RANKL expression in their gastric mucosa decreased after the treatment (**d**–**f**, **j**–**l**). Immunostained specimens were assessed by pathologists blinding to clinical features.

For patient2 and Patient3 with severe arthralgia, we treated them with celecoxib, a nonsteroidal anti-inflammatory drug used to treat pain or inflammation through specifically inhibiting COX-2 and reducing PGE_2_ generation, for three months^34, 35^. According to the patients’ response, their arthralgia was partly relieved. Moreover, endoscopy as well as histological examination (Fig. 4) showed that although the proliferation of mucous glands still existed, both the inflammation of the gastric mucosa and the intracellular mucus were reduced as expected (Fig. 4 Patient2 g h, Patient3 o p). Moreover, IL-6, TNFα and RANKL expression in gastric mucosa of patient2 and patient3 were also decreased after them receiving celecoxib treatment for three months (Fig. 5 Patient2 d-f, Patient3 j-l). And these declines were obvious in IOD (Table S2).

## Discussion

Homozygous variants and compound heterozygous variants in *HPGD* and *SLCO2A1* were reported to cause PDP^3, 4^. In this study, we identified four novel variants in *SLCO2A1*, among which c.96 + 4 A > C is predicted to cause an abnormal splicing event by in silico analysis. Unfortunately, we have not been able to provide corresponding data to prove it because of the unavailability of the patient’s RNA. Further studies are needed to assess the effects of this splice site variant. Localized within highly homologues regions, both residue Tyr357 and Pro469 are highly conserved across species. *In silico* analysis indicated that c.1069T > C and c.1406C > T have high probability to affect the protein function (Table S3). Correspondently, the MAF (Minor Allele Frequency) are low in Exome Aggregation Consortium (EXAC: http://exac.broadinstitute.org/) and 1000 Genomes Project (1000 G: http://browser.1000genomes.org). According to the ACMG guideline^28^, we classified these two novel variants as likely pathogenic. The fourth novel variant c.1771C > T in *SLCO2A1*, which wasn’t recorded in either EXAC or 1000 G, replaces the Arg591 with a stop codon. Early in 2013, a *SLCO2A1* null variant c.1807C > T (p.R603X), resulting in a loss of the last transmembrane domain of PGT, was found in a patient with PDP^36^. Comparing with it, c.1771C > T causes a loss of an even bigger fragment, including an important structure for PGT function as a transporter^37^. Consequently, we assumed that the c.1771C > T variant would affect its function as well. These variants combined with another *SLCO2A* variant were considered as the cause of PDP for patient4 (Fig. 1c II-1), patient5 (Fig. 1d II-1) and his little sister (Fig. 1d II-2) and patient7 (Fig. 1f II-4).

Gastric system condition in PDP patients was rarely concerned. But early in 1983, Lam *et al*. described two PDP brothers who had hypertrophic gastritis^16^. In 2001, Zrinka Jajic *et al*. showed gastroscopy test was positive (ulcer and hypertrophic gastritis) in all 21 PDP patients (100%). But this report did not attract the attention of researchers. About 261 PDP patients were reported between 2001 and 2017. Gastroscopy was performed only in 25 patients (9.58%) and 16 patients were showed to be positive (64%): 7 patients had gastric mucosa hyperplasia and 4 patients received histological examination, 3 patients had gastritis, 4 patients had gastric polyps and 2 patients had gastric carcinoma. So a large proportion of PDP patients did not receive the digestive system examination. In our study, we systematically revealed the gastric mucosa hyperplasia in all of eight PDP patients. We believe that the incidence of digestive system abnormities in PDP, especially the gastric mucosa hyperplasia, is much higher than it have been reported^20^. So gastric mucosa hyperplasia could be regarded as a common manifestation of PDP and related examinations were advised in every PDP patient.

Although the pathogenesis of PDP is currently unknown, causal variants of *HPGD* and *SLCO2A1* increase PGE_2_ level^3, 4, 7, 38^. PGE_2_ has a conflicting effect on gastric mucosa. As a protection mechanism, physiological PGE_2_ contributes to the recovery of gastric mucosa injury through promoting gland cell regeneration and other effects^39^. But in pathological condition, elevated PGE_2_ could accelerate gastric mucosa cell proliferation and plays an important role in the development of tumors through various mechanisms^40, 41^. Consistently, we observed in our study that all eight PDP patients had different degree of gastric mucosa hyperplasia from combined results of endoscopy and histological examination. What’s more, this would suggest an increased risk of gastric cancer in PDP patients. And PDP patients do tend to develop gastric tumor at a relatively young age^17, 20, 39, 40^. Therefore, it is possible that high PGE_2_ level in PDP patients might result in gastric mucosa hyperplasia even hyperplasic tumor in a similar way. And the majority of PGE_2_ formed *in vivo* is derived from COX-2 and COX-2 also contributes significantly to the overproduction of PGE_2_^42^. Thus, we treated patient2 and patient3 who had severe arthralgia with celecoxib. After three months, their arthralgia and gastric mucosa hyperplasia were partly relieved and no side-effects were complained. Celecoxib is a NSAID which specifically inhibit COX-2 and reduce PGE_2_ generation. Our results support published individual case reports that specific COX-2 inhibitor NSAIDs treatment, such as celecoxib, etoricoxib etc, could relief PDP patients’ suffer and could not worry about its side-effects on digestive system^13, 43, 44^.

Other publications described inflammation and bone metabolism were also involved in PDP^23, 45^. IL-6, TNFα and RANKL are three of the most important factors. IL-6 is a pro-inflammatory cytokine that produces multifunctional effects, such as proliferation and differentiation and act as a key player during inflammation^46, 47^. TNFα is also a pro-inflammatory cytokine associated with pleiotropic effects on different cell-types. It is produced by lots of cell types involved in inflammation. TNFα can stimulate PGE_2_ synthesis and can also increase RANKL expression^23, 48^. RANKL is a member of the TNF superfamilies. The main function of RANKL is to stimulate bone resorption through osteoclastogenesis and the activation of multinucleated mature osteoclasts^49, 50^. RANKL expression can be induced by PGE_2_, IL-6 and TNFα^51^. High level of IL-6, TNFα and RANKL in serum and synovial fluid was observed in PDP patients^21–23^. IL-6, TNFα and RANKL were regarded as bone turnover markers and bone resorption and arthritis in PDP were probably mediated by them^21, 52^. Our results showed IL-6, TNF α and RANKL expression were elevated in hypertrophic gastric mucosa of PDP patients (Fig. 3, Fig. S2). Moreover, their expressions were in keeping with the severity of gastric mucosa hyperplasia (Fig. 3) and decreased after the patients received specific COX-2 inhibitor (celecoxib) treatment (Fig. 5, Table S2). We speculate that PGE_2_, IL-6, TNFα and RANKL are involved in the molecular mechanisms of gastric mucosa hyperplasia in PDP.

Further researches are needed to more completely elucidate the association between gastric mucosa hyperplasia, PGE_2_, IL-6, TNFα and RANKL increase in PDP.

## Conclusion

Here, we identified four novel variants in the *SLCO2A1* gene and demonstrated that gastric mucosa hyperplasia might be a common clinical manifestation of PDP and celecoxib would be a considerable treatment for PDP patients. We also revealed that IL-6, TNFα and RANKL may play important roles in the molecular mechanisms of gastric mucosa hyperplasia in PDP for the first time.

## Electronic supplementary material

Supplementary Information
