# Supplementary material for: Interleukin-6, tumor necrosis factor-alpha and receptor activator of nuclear factor kappa ligand are elevated in hypertrophic gastric mucosa of pachydermoperiostosis
Source: Sci Rep. 2017 Aug 29;7:9686. doi: 10.1038/s41598-017-09671-7 (PMC5574921; doi:10.1038/s41598-017-09671-7)
Supplement: Supplementary file 1 — Supplementary Information [file 41598_2017_9671_MOESM1_ESM.pdf]

Title: Interleukin-6, tumor necrosis factor-alpha and receptor activator of nuclear factor kappa ligand are elevated in hypertrophic gastric mucosa of pachydermoperiostosis

Hui Huang[1,2], Yongjun Wang[2], Yong Cao[3], Boda Wu[1,2], Yonggui Li[1,2], Liangliang Fan[4], Zhiping Tan[4], Yi Jiang[5], Jianguang Tang[6], Jianzhong Hu[3*], Xiaoliu Shi[1, 2*]

[1]Department of Medical Genetics, The Second Xiangya Hospital, Central South University, Changsha, Hunan410011, China;

[2]Department of Gastroenterology, The Second Xiangya Hospital, Central South University, Changsha, Hunan410011, China;

[3]Department of Spine Surgery, Xiangya Hospital, Central South University, Changsha, Hunan 410008, China;

[4] The Thoracic Surgery Research Room, The Second Xiangya Hospital, Central South University, Changsha, Hunan410011, China;

[5]Department of Pathology, The Second Xiangya Hospital, Central South University, Changsha, Hunan410011, China;

[6]Department of Neurology, The Second Xiangya Hospital, Central South University, Changsha, Hunan410011, China.

[*]These authors contributed equally to the paper.

Corresponding author: Xiaoliu Shi, room1308, science building, The Second Xiangya Hospital, Central South University, No.139,

Renmin Middle Road, Furong District, Changsha, Hunan410011, P.R. China. Phone: +86 731 85292206, Fax: +86 731 85292205, e-mail: shixl6@csu.edu.cn.

Co-corresponding author: Jianzhong Hu. Department of Spine Surgery, Xiangya Hospital, Central South University, Changsha，410008, Hunan, P.R. China；Phone：+86-731-84327327, Fax：+86-731-84327332, email：jianzhonghu@hotmail.com.

Supplementary Legends

Fig S1. The symbolic manifestations of patients with pachydermoperiostosis.

PDP patients with thickening and furrowing facial features (a), periosteal thickness (white arrows) (b), severely swollen knee joints (c), typical digital clubbing (d), and acroosteolysis (black arrows) (e). a and b were from patient2. c was from patient4. d and e were from patient8.

Fig S2. Comparison of IOD in gastric mucosa of PDP patients and controls.

IOD, integrated optical density. SPSS13.0 software was employed. Data which were normally distributed were expressed as mean ± standard error of the mean (S.E.M.). Statistical evaluation was performed using Mann–Whitney U-test to differentiate non-parametric means of different groups. IOD of IL-6, TNFα and RANKL expressions in gastric mucosa of PDP patients (n=8) were statistically higher than that in the controls (n=4) (* $p<0.05$, ** $p<0.01$).

Table S1. The primers of coding regions in *SLCO2A1* and *HPGD*.

Table S2. Comparison of IOD in gastric mucosa of patient2 and patient3 before/after treatment.

IOD, integrated optical density. Data were expressed as mean ± standard error of the mean (S.E.M.). IOD in gastric mucosa of patients declined after them receiving the celecoxib treatment.

Table S3. Damaging effects of variants detected in pachydermoperiostosis patients predicted by online software.

P, patient. N, the variant has been reported. Y, the variant is reported by us for the first time. WT, wild type.

1. Zhang, Z, He, J.W, Fu, W.Z et al. *Mutations in the SLCO2A1 gene and primary hypertrophic osteoarthropathy: a clinical and biochemical characterization.* J Clin Endocrinol Metab, 2013. **98**(5): p. 923-33.
2. Niizeki, H, Shiohama, A, Sasaki, T et al. *The novel SLCO2A1 heterozygous missense mutation p.E427K and nonsense mutation p.R603* in a female patient with pachydermoperiostosis with an atypical phenotype.* Br J Dermatol, 2014. **170**(5): p. 1187-9.
3. Diggle CP, Parry DA, Logan CV et al. *Prostaglandin transporter mutations cause pachydermoperiostosis with myelofibrosis.* Hum Mutat, 2012. **33**(8): p. 1175-81.
4. Zhang, Z, He, J.W, Fu, W.Z et al. *A novel mutation in the SLCO2A1 gene in a Chinese family with primary hypertrophic osteoarthropathy.* Gene, 2013. **521**(1): p. 191-4.
5. Erken, E, Koroglu, C, Yildiz, F et al. *A novel recessive 15-hydroxyprostaglandin dehydrogenase mutation in a family with primary hypertrophic osteoarthropathy.* Mod Rheumatol, 2015. **25**(2): p. 315-21.

Figure S1

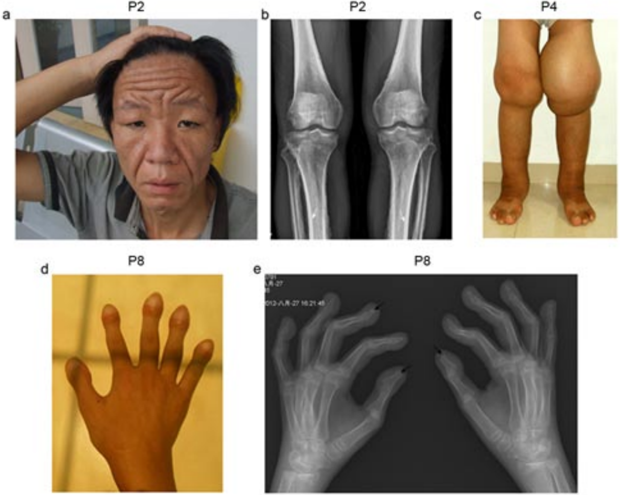

Figure S2

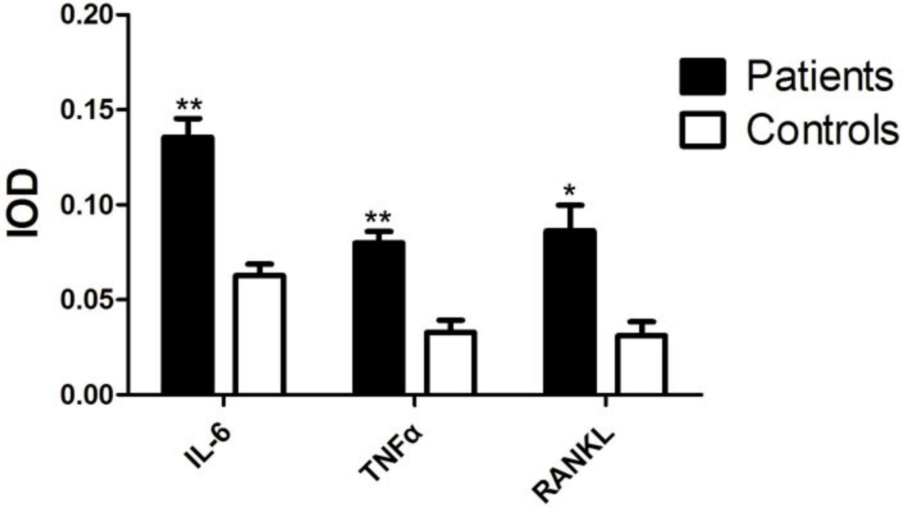

TableS1. The primers of coding regions in *SLCO2A1* and *HPGD*.

Target	Forward primer sequence(5'-3'	Reward primer sequence(3'-5')
SLCO2A1 exon1	gaatctcctccggccact	ggctccggcagacagaag
SLCO2A1 exon2	cactgggccacatatcacag	ctgttacccggcagaaagag
SLCO2A1 exon3	ggagatggagacccagaagg	gcacactttcctgaacaaacc
SLCO2A1 exon4	caggaaccatgtcccatttg	acacagctgggaggtaatgg
SLCO2A1 exon5	acaggtgtgggcttatcagg	cagcagcttgttcctcacag
SLCO2A1 exon6	cctctgggaagaccaatagc	tggaggtctcctgatccttg
SLCO2A1 exon7	ggaaatgcaggtgctgtttg	tctgctcctactgtcccttac
SLCO2A1 exon8	ccctgtggtgttgtgtgc	ctgactggaaggacaggag
SLCO2A1 exon9	gcctggcaagcagtaaattg	tgcttgaacctgggagaatc
SLCO2A1 exon10	aaatggagagatgccgtgac	cccagggtagggaggtagag
SLCO2A1 exon11	ttgcccaaacagtgacagag	cctgcaatgaggagctcag
SLCO2A1 exon12	tagagcattcagcccaggtg	cctcaagcaatctgggaaac
SLCO2A1 exon13	gcccgtgtatctccactctg	tggcccttcatgttctcttc
SLCO2A1 exon14	cctgcttccctacagctttg	gggtacacagtggcccttag
HPGD exon1	gctggcttgacagtttcctc	agtctcggagtgtgtgggc
HPGD exon2	gtgtgtttattgtttgtccgtc	acgttcccagttgacagattg
HPGD exon3	cctctcatggcataggacatg	gtttccatgactccaagaacc
HPGD exon4	gtattccttttctcacttatgc	tgaagatttgtttttgtggtcc
HPGD exon5	gagtttcacaaagctatctgg	tgagatatgacggttgttgtag
HPGD exon6	gaaactgctgaaacctacaac	ctgtataagcttatttcttccc
HPGD exon7	cacatttccctataacatgttc	agctatggctaacacataagc

Table S2. Comparison of IOD in gastric mucosa of patient2 and patient3 before/after treatment.

		IOD（Integrated Optical Density）（Mean±S.E.M）					
		IL-6		TNFα		RANKL	
		Before treatment	After treatment	Before treatment	After treatment	Before treatment	After treatment
Patients	patient2	0.1642±0.0459	0.0646±0.0104	0.0989±0.0166	0.0088±0.001	0.1565±0.0008	0.0195±0.0017
	patient3	0.1279±0.0328	0.0634±0.0136	0.0639±0.0158	0.0227±0.0110	0.0929±0.0279	0.0334±0.0119

TableS3.Damaging effects of variants detected in pachydermoperiostosis patients predicted by online soft wares.

Family	Gene	Patient	Genotype	Protein	Novel	Mutation taster	Polyphen2	SIFT	HSF
1	*SLCO2A1*	P1(Fig1 a II-2)	c.1106G>A	p.G369D	N[1]	Disease causing (Score＞0.9999)	Probably Damaging (Score:1.000)	Damaging (Score:0)	–
		P2(Fig1 a II-4)	c.1106G>A	p.G369D	N	Disease causing (Score＞0.9999)	Probably Damaging (Score:1.000)	Damaging (Score:0)	–
2	*SLCO2A1*	P3(Fig1 b II-2)	c.1106G>A	p.G369D	N	Disease causing (Score＞0.9999)	Probably Damaging (Score:1.000)	Damaging (Score:0)	–
			c.1807C>T	p.R603X	N[2]	Disease causing (Score:1)	–	–	–
3	*SLCO2A1*	P4(Fig1 c II-1)	c.941-1G>A	p.?	N[3]	Disease causing (Score:1)	–	–	Broken WT Acceptor Site
			c.1771C>T	p.R591X	Y	Disease causing (Score:1)	–	–	–
4	*SLCO2A1*	P5(Fig1 dII-1)	c.1406C>T	p.P469L	Y	Disease causing (Score＞0.9999)	Probably Damaging (Score:1.000)	Damaging (Score:0)	–
			c.1602C>A	p.N534K	N[4]	Disease causing (Score＞0.9999)	Probably Damaging (Score:0.984)	Damaging (Score:0.04)	–
5	*SLCO2A1*	P6(Fig1 e II-1)	c.611C>T	p.S204L	N[5]	Disease causing (Score＞0.9999)	Probably Damaging (Score:1.000)	Damaging (Score:0)	–
6	*SLCO2A1*	P7(Fig1 f II-4)	c.96+4A>C	p.?	Y	–	–	–	Broken WT Donor Site
			c.1069T>C	p.Y357H	Y	Disease causing (Score＞0.9999)	Probably Damaging (Score:0.999)	Damaging (Score:0)	–
7	*HPGD*	P8(Fig1 g II-1)	c.310-311delCT	p.L104Afs*3	N[5]	Disease causing (Score:1)	–	–	–
